# Anesthetic Considerations in Pediatric Craniosynostosis Repair: Multidisciplinary Insights and Challenges

**DOI:** 10.7759/cureus.87313

**Published:** 2025-07-05

**Authors:** Kathiravan K, Ranjan RV, Eshvanthni V, Rajarajacholan D

**Affiliations:** 1 Anesthesiology and Critical Care, Sri Manakula Vinayagar Medical College and Hospital, Puducherry, IND

**Keywords:** acute blood loss, craniosynostosis review, craniosynostosis surgery, difficult airway management, pediatric critical care

## Abstract

Craniosynostosis is a congenital condition marked by the premature fusion of cranial sutures, resulting in abnormal skull shapes and potential neurological complications. This report describes the anesthetic management of a two-year-nine-month-old male diagnosed with sagittal craniosynostosis who underwent calvarial reconstruction with fronto-orbital advancement. The case presented notable anesthetic challenges, including airway management, significant intraoperative blood loss, and the need for careful postoperative sedation. A multidisciplinary approach was key to achieving a successful outcome.

## Introduction

Craniosynostosis is characterized by the premature fusion of one or more cranial sutures and occurs in approximately 1 in 2,000 to 2,500 live births [1]. While it often presents as an isolated condition, up to 40% of cases are associated with syndromes such as Pfeiffer, Apert, Crouzon, Muenke, Saethre-Chotzen, and Carpenter syndromes [2]. Craniosynostosis can be classified as either simple, affecting a single suture, or complex, involving multiple sutures. Its clinical presentation varies depending on the affected sutures, with common types including scaphocephaly, plagiocephaly, trigonocephaly, and brachycephaly [3]. The premature fusion restricts skull growth perpendicular to the involved suture, leading to compensatory growth along other sutures to accommodate brain development. This results in distinct cranial deformities [4].

Early surgical intervention is advised to support normal brain development and improve cosmetic outcomes, though the benefits must be balanced against the risks of major surgery and prolonged anesthesia in infants. Surgery is typically performed between six and 12 months of age, a period during which skull bones are more amenable to reshaping. Minimally invasive endoscopic techniques are often preferred to reduce surgical morbidity. Preoperative assessment of the airway and cardiac function is critical, particularly in syndromic cases where obstructive sleep apnea (OSA) and respiratory complications are common. Anesthetic management must account for current respiratory infections, congenital heart disease, difficult IV access, challenges with ventilation and intubation, patient positioning, raised intracranial pressure (ICP), and the risk of significant intraoperative blood loss. Outcomes are influenced by surgical technique, perioperative management, and the prompt identification and treatment of complications. Early detection of perioperative warning signs is essential to improve prognosis and minimize long-term sequelae [3].

This case report highlights the anesthetic challenges and considerations encountered in the perioperative management of a pediatric patient undergoing surgical correction for craniosynostosis.

## Case presentation

Case background

A two-year-nine-month-old male child with a history of microcephaly was referred for surgical correction of craniosynostosis. The child exhibited developmental delays, with notable difficulties in motor skills and communication. He also had a documented seizure disorder and a history of recurrent upper respiratory tract infections that had required nebulization.

Physical examination

**Figure 1:**
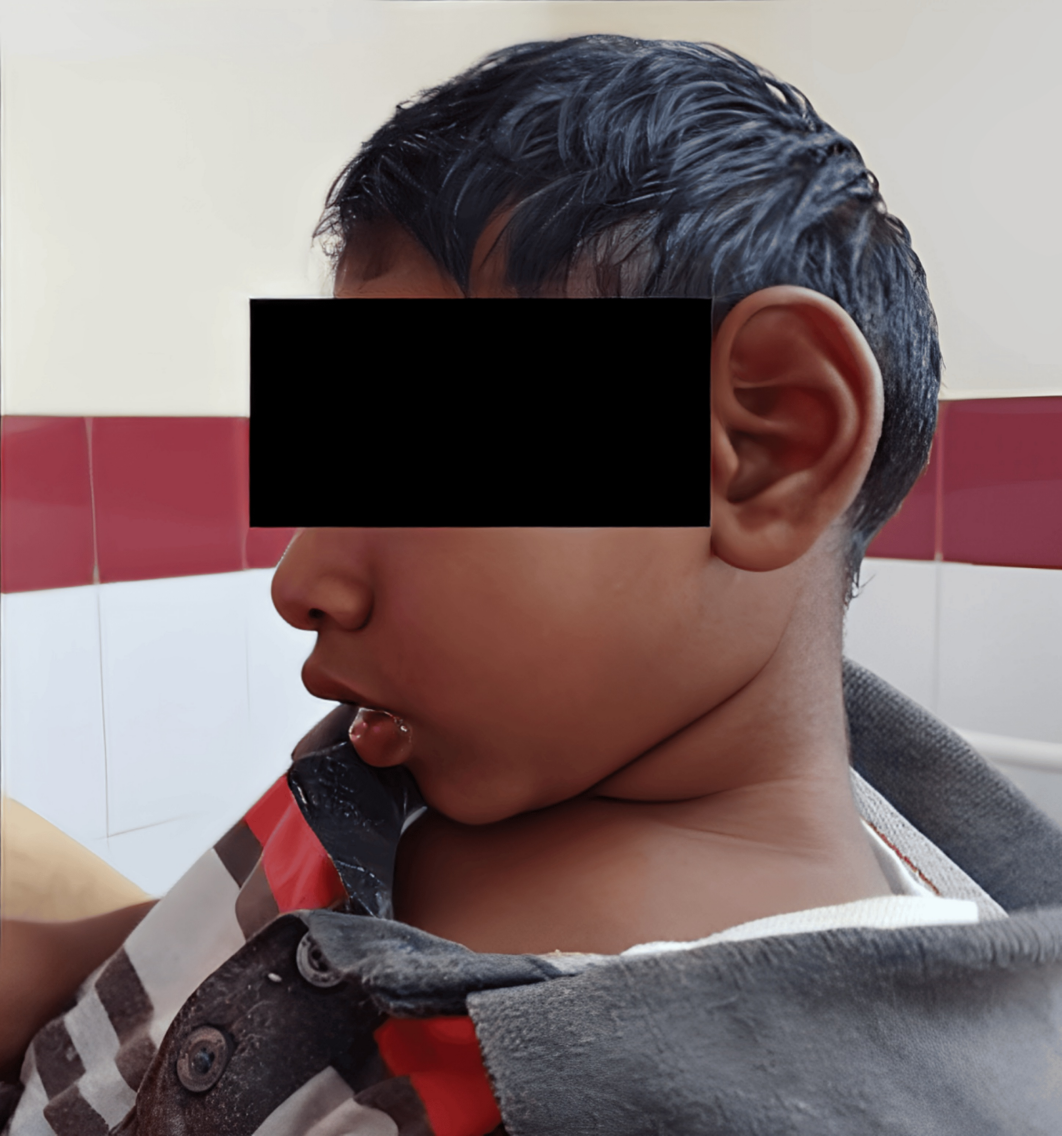
Clinical photograph showing dysmorphic facial features

On examination, the child was alert but hyperactive. Dysmorphic facial features were observed, including a boat-shaped head, retrognathia, and mild midface hypoplasia (Figure 1). His head circumference measured 34.5 cm, and his height and weight were 82 cm and 9.5 kg, respectively; all three measurements fell below the third percentile. Neurological examination revealed no focal deficits, but developmental assessment indicated delayed gross motor milestones and impaired expressive speech.

Preoperative investigations

Routine laboratory investigations, including complete blood count, renal function tests, serum electrolytes, coagulation profile, and blood grouping and typing, were conducted and found to be within normal limits. EEG revealed generalized slowing, predominantly over the bilateral frontal and central regions, suggestive of diffuse cerebral dysfunction.

Imaging studies

**Figure 2:**
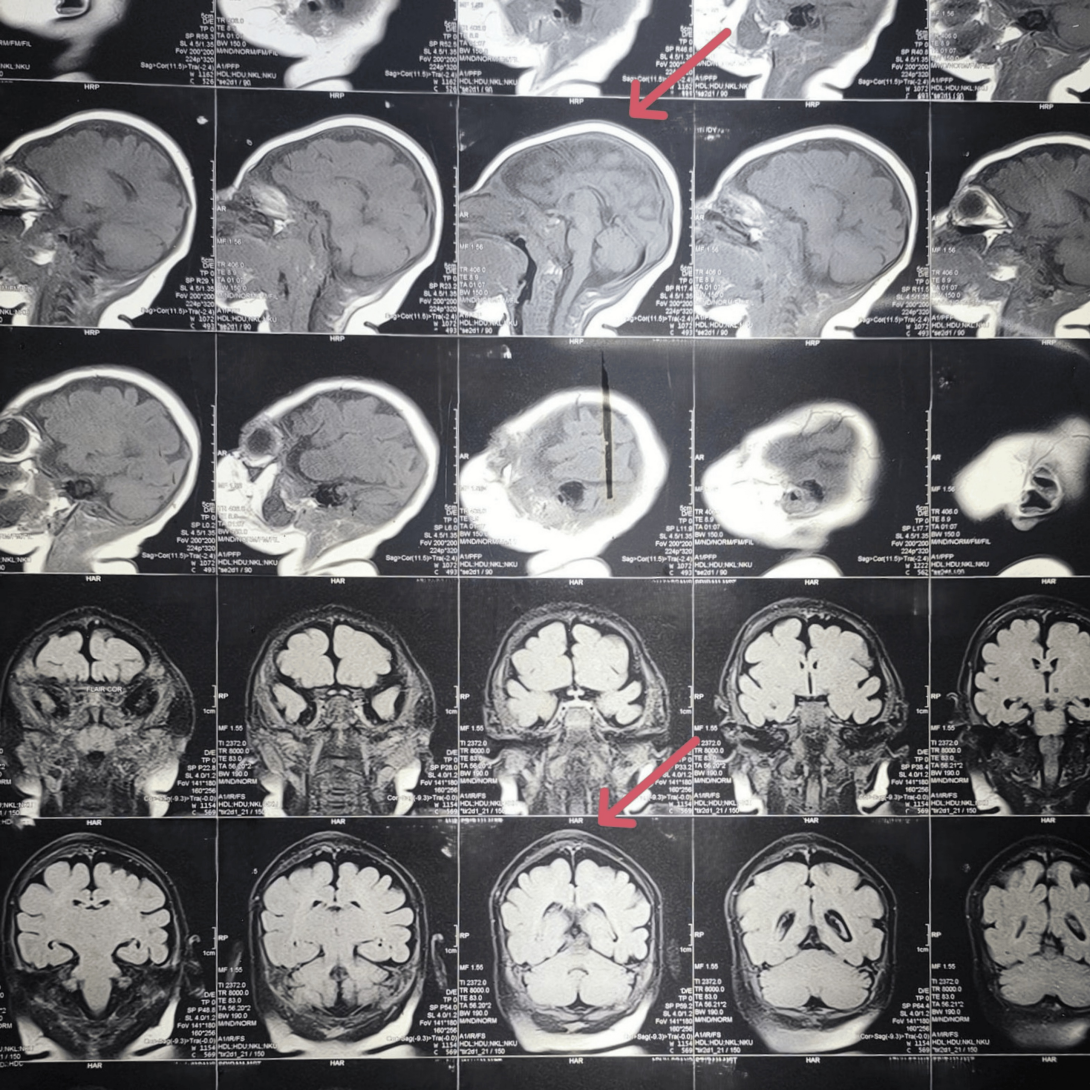
MRI of the brain showing findings suggestive of microcephaly, premature fusion of cranial sutures, and mild diffuse cerebral atrophy

**Figure 3:**
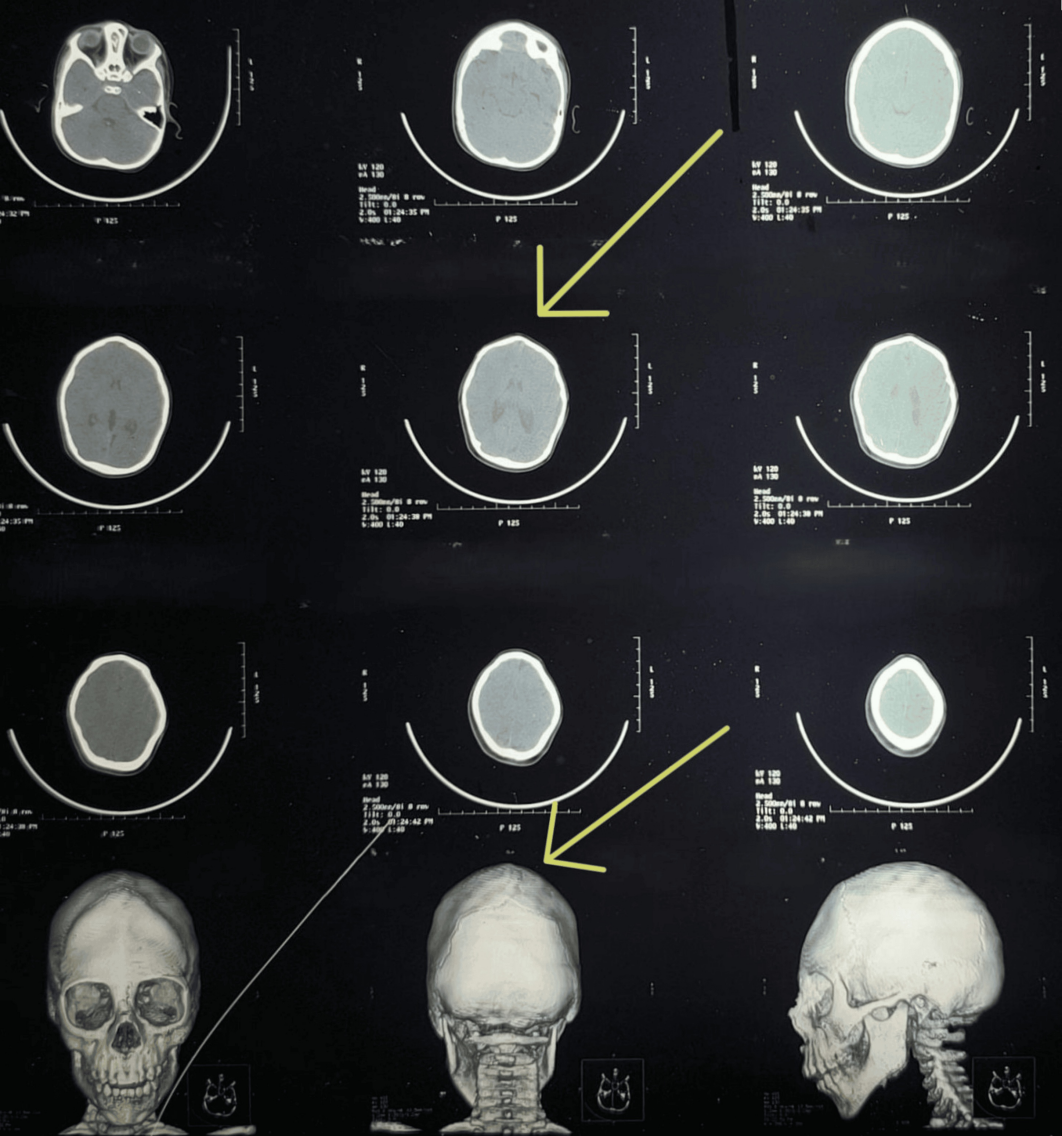
CT scan of the brain demonstrating premature fusion of the sagittal suture

MRI of the brain demonstrated microcephaly, premature fusion of cranial sutures, and mild diffuse cerebral atrophy (Figure 2). CT of the brain showed craniosynostosis with premature fusion of the sagittal suture, resulting in a mildly elongated skull consistent with scaphocephaly (dolichocephaly) (Figure 3).

Anesthetic challenges

Several anesthetic challenges were anticipated in this case, and the child was classified as ASA Physical Status III. The foremost concern was difficult airway management due to craniofacial dysmorphisms, which posed a significant risk during intubation. To address this, the anesthesia team was well prepared with a range of airway management tools, including a size 3 video laryngoscope blade, fiberoptic bronchoscope (OD 3.8 mm, ID 1.2 mm), small bougie, stylet, and a tracheostomy kit.

Another major concern was intraoperative blood loss. Craniosynostosis surgeries are often associated with substantial bleeding, necessitating the preoperative arrangement of adequate blood products. In this case, considerable blood loss was anticipated, and appropriate preparations were made.

Hypothermia was also a critical issue, given the extensive scalp exposure during surgery, which could lead to significant heat loss. To prevent this, both active and passive warming strategies were employed, including forced-air warming blankets, fluid warmers, cotton roll insulation, and prewarmed IV fluids. The operating theater temperature was maintained at 24°C throughout the procedure.

Postoperative pain management required special attention. Providing effective analgesia while minimizing the risk of respiratory depression was essential, particularly given the patient’s young age. A balanced approach was planned to ensure comfort and safety during the postoperative period.

Anesthetic management

Preoperative Preparation

A thorough preanesthetic assessment was conducted, and informed consent was obtained from the parents, who were made aware of the risks associated with surgery and anesthesia. An emergency intubation cart was prepared, and blood products were reserved in anticipation of transfusion needs.

Induction of Anesthesia

On the day of surgery, a 22-G IV line was secured using a Venflon in the left hand at 5:00 AM while the child was sleeping, facilitated by the application of EMLA cream. Premedication included midazolam (0.2 mg IV) and propofol (10 mg IV) in the holding area to facilitate smooth parental separation.

Upon arrival in the operating room, standard ASA monitors were applied, including ECG, non-invasive blood pressure, pulse oximetry, capnography, a precordial stethoscope, and temperature monitoring. The difficult airway cart was kept ready, containing a small bougie, stylet, size 3 video laryngoscope blade, fiberoptic bronchoscope (OD 3.8 mm, ID 1.2 mm), and tracheostomy kit.

Anesthesia was induced using propofol (10 mg IV) and fentanyl (20 mcg IV). Neuromuscular blockade was achieved with atracurium (5 mg IV). Intubation was successfully performed using a 4 mm ID cuffed South Pole Ring-Adair-Elwyn (RAE) tube, and controlled ventilation was established using a mixture of oxygen and sevoflurane.

Under aseptic precautions, the right internal jugular vein was cannulated with a 5-F triple-lumen catheter, and the left radial artery was cannulated using a 24-G cannula for invasive hemodynamic monitoring.

Intraoperative Management

Analgesia was maintained with a continuous infusion of fentanyl at 5 mcg/hr, supplemented with a single IV dose of morphine (1 mg). Fluid management followed the Holliday-Segar formula, with maintenance requirements met using 400 mL of Ringer’s lactate and 100 mL of 10% dextrose.

Intraoperative blood loss was estimated at approximately 200 mL, assessed using suction canister measurements, gauze weight, and evaluation of the surgical field. Blood and fluid losses were managed with the transfusion of 200 mL of packed red blood cells and 100 mL of fresh frozen plasma. Central venous pressure was continuously monitored via the central venous catheter to guide volume resuscitation. A single arterial blood gas analysis was performed during the procedure.

**Figure 4:**
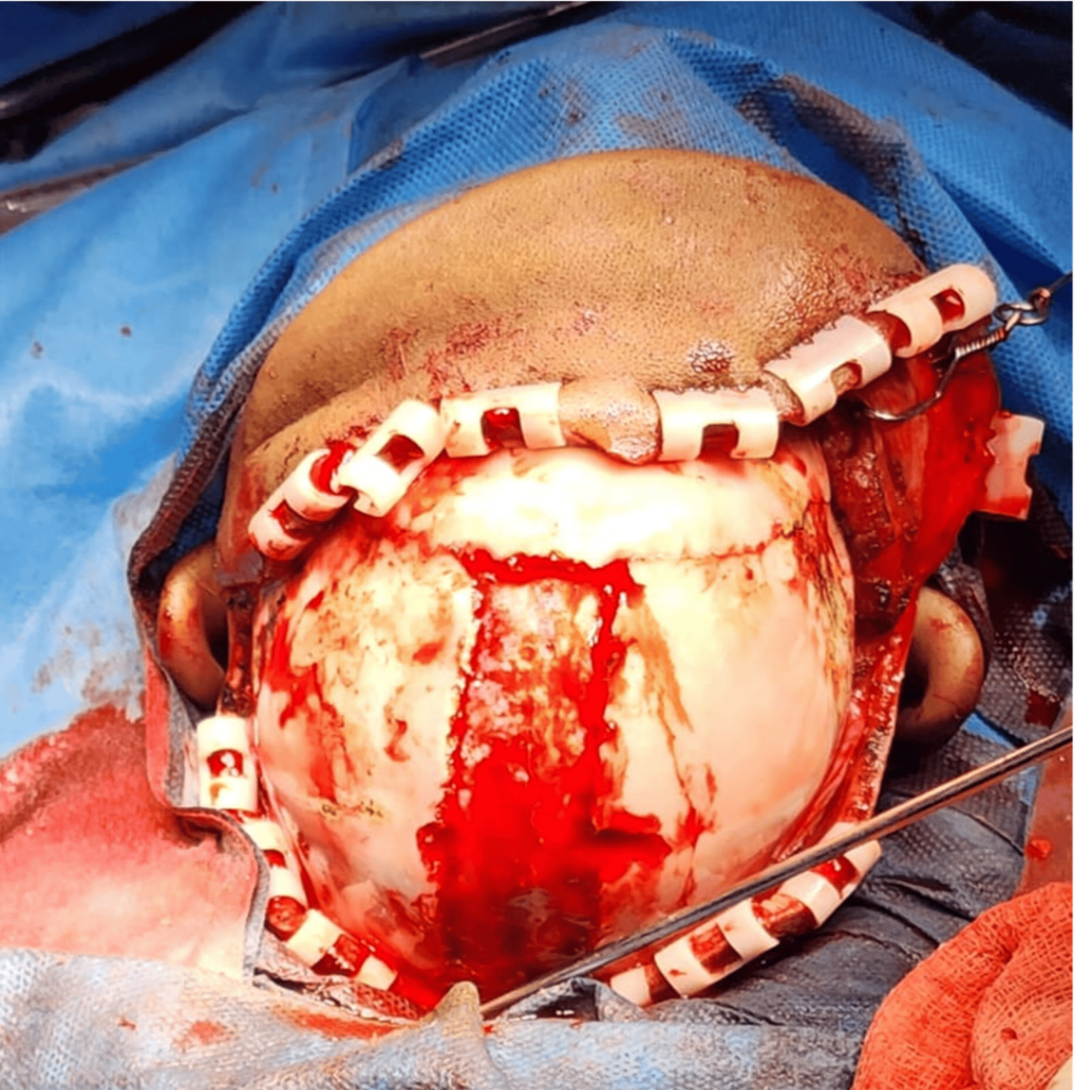
Intraoperative image depicting calvarial reconstruction surgery

To reduce surgical blood loss, tranexamic acid was administered slowly at a dose of 10 mg/kg. The child underwent close hemodynamic monitoring throughout the five-hour procedure. Despite significant blood loss, effective fluid and transfusion management maintained hemodynamic stability (Figure 4).

Postoperative Care

Following surgery, the RAE South Pole endotracheal tube was replaced with a standard polyvinyl chloride endotracheal tube, and the patient was transferred to the pediatric ICU for elective mechanical ventilation and close monitoring. Initially, the child was managed on synchronized intermittent mandatory ventilation mode. Despite sedation with fentanyl at 5 mcg/hr, the child remained agitated, necessitating an increase in the fentanyl infusion to 10 mcg/hr. To manage breakthrough pain, a 250 mg paracetamol suppository and a 1 mg IV bolus of morphine were administered. The fentanyl infusion was then gradually tapered and discontinued by 6:00 AM the following morning. The patient was subsequently transitioned to continuous positive airway pressure and was successfully extubated at 10:00 AM.

## Discussion

Craniosynostosis is a multifactorial condition typically arising from a combination of genetic and environmental factors. Early surgical intervention is now the standard approach for managing most types of craniosynostosis. Surgical options include reconstructive procedures, as in the present case, as well as endoscopic techniques. The primary goals of anesthetic management are to minimize blood loss, avoid hypothermia, and manage potential airway difficulties [5]. Children with craniosynostosis may require multiple surgeries to address complications such as elevated ICP, severe exophthalmos, OSA, and craniofacial deformities and to prevent neurological and psychosocial consequences.

Preoperative evaluation must be individualized, as disease severity and clinical presentation vary among patients. Airway difficulties are commonly encountered and may complicate both mask ventilation and direct laryngoscopy. The preanesthetic assessment should focus on optimizing respiratory function, especially since airway complications such as wheezing are frequently observed in syndromic craniosynostosis cases, including Apert syndrome, which may delay surgical procedures. Premedication must be carefully titrated, as excessive sedation can result in airway obstruction [6].

In a study by Lionel et al., 76% of patients with syndromic craniosynostosis experienced difficult airway management, requiring awake fiberoptic bronchoscopy or video laryngoscopy for intubation [7]. Another study reported a difficult airway incidence of approximately 58% [8]. In our case, endotracheal intubation was successfully performed using a video laryngoscope and a 4 mm ID cuffed RAE tube (South Pole design).

Craniosynostosis surgeries are also associated with significant blood loss. Therefore, preoperative assessment of hemoglobin levels, blood type and screen, and ensuring the availability of cross-matched blood products are essential for maintaining hemodynamic stability. Rapid and substantial bleeding may occur from the scalp and bony structures, increasing the risk of intraoperative instability. In our case, we maintained normothermia, administered prophylactic tranexamic acid, tolerated lower mean arterial pressures, and kept blood products ready to mitigate the risk.

Pediatric patients undergoing craniosynostosis repair are also prone to hypothermia, particularly those with Apert syndrome, who tend to exhibit excessive sweating. Hypothermia can exacerbate blood loss, lead to metabolic acidosis, and result in myocardial depression [9]. To prevent this, the patient’s temperature was carefully controlled: the extremities were covered, the operating room temperature was maintained at 24°C, forced-air warming blankets were used, and all fluids were administered through a warming device. Maintaining normothermia has been shown to significantly reduce intraoperative blood loss compared to hypothermic patients.

A multidisciplinary approach involving neurosurgeons, pediatricians, anesthesiologists, and nursing staff was crucial in achieving optimal patient outcomes. Attention to perioperative detail, such as managing temperature, airway, and hemodynamics, can significantly reduce the risk of complications.

Postoperatively, the patient was electively ventilated to ensure correction of temperature and electrolyte imbalances and to manage pain using high-dose opioids. Once these factors were stabilized, the patient was safely extubated under controlled conditions and closely monitored thereafter. Continuous monitoring and timely pain management played essential roles in facilitating recovery and ensuring patient comfort.

## Conclusions

This case highlights the complexity of anesthetic management in a pediatric patient undergoing craniosynostosis repair. Through meticulous preoperative planning, vigilant intraoperative management, and comprehensive postoperative care, the anesthesia team successfully addressed the multifaceted challenges of the procedure. Multidisciplinary collaboration was integral in achieving a favorable outcome. This report underscores the importance of tailored anesthetic strategies and proactive perioperative management to optimize safety and recovery in craniosynostosis surgeries.
